# Differences in clinical features and mortality in very old unvaccinated patients (≥ 80 years) hospitalized with COVID-19 during the first and successive waves from the multicenter SEMI-COVID-19 Registry (Spain)

**DOI:** 10.1186/s12877-022-03191-4

**Published:** 2022-06-30

**Authors:** Jose-Manuel Ramos-Rincon, Lidia Cobos-Palacios, Almudena López-Sampalo, Michele Ricci, Manel Rubio-Rivas, Maria-Victoria Nuñez-Rodriguez, Rodrigo Miranda-Godoy, Maria-Eugenia García-Leoni, Rosa Fernández-Madera-Martínez, Gema-María García-García, Jose-Luis Beato-Perez, Daniel Monge-Monge, Uxua Asín-Samper, Marta Bustamante-Vega, Isabel Rábago-Lorite, Santiago-Jesús Freire-Castro, Jose-Pablo Miramontes-González, Jeffrey-Oskar Magallanes-Gamboa, José-Nicolás Alcalá-Pedrajas, Miriam García-Gómez, Verónica Cano-Llorente, Francisco-Javier Carrasco-Sánchez, Jesús Martinez-Carrilero, Juan-Miguel Antón-Santos, Ricardo Gómez-Huelgas, the SEMI-COVID-19 Network

**Affiliations:** 1 Department of Clinical Medicine, Miguel Hernández University of Elche, Ctra N332 s/n, 03550 Sant Joan d’Alacant, Alicante, Spain; 2 Department of Internal Medicine, Instituto de Investigación Biomédica de Málaga (IBIMA), Regional University Hospital of Málaga & University of Málaga, Málaga, Spain; 3 Internal Medicine Department, Bellvitge University Hospital, , Barcelona, L’Hospitalet de Llobregat Spain; 4 Internal Medicine Department, Costa del Sol Hospital, Marbella, Spain; 5 Internal Medicine Department, 12 de Octubre University Hospital, Madrid, Spain; 6 Internal Medicine Department, Gregorio Marañon University Hospital, Madrid, Spain; 7 Internal Medicine Department, Cabueñes University Hospital, Gijón, Spain; 8 Internal Medicine Department, Badajoz University Hospital Complex, Badajoz, Spain; 9 Internal Medicine Department, Albacete University Hospital Complex, Albacete, Spain; 10 Internal Medicine Department, Segovia Hospital Complex, Segovia, Spain; 11 Internal Medicine Department, Miguel Servet University Hospital, Zaragoza, Spain; 12 Internal Medicine Department, La Princesa University Hospital, Madrid, Spain; 13 Internal Medicine Department, Infanta Sofía University Hospital, S. S. de los Reyes, Madrid, Spain; 14 Internal Medicine Department, A Coruña University Hospital Complex, A Coruña, Spain; 15 Internal Medicine Department, Rio Hortega University Hospital, Valladolid, Spain; 16 Internal Medicine Department, Nuestra Señora del Prado Hospital, Talavera de La Reina, Toledo, Spain; 17 Internal Medicine Department, Pozoblanco Hospital, Pozoblanco, Córdoba, Spain; 18 Internal Medicine Department, Alfredo Espinosa Hospital, Urduliz, Vizcaya Spain; 19 Internal Medicine Department, Virgen de La Salud University Hospital, Toledo, Spain; 20 Internal Medicine Department, Juan Ramón Jiménez University Hospital, Huelva, Spain; 21 Internal Medicine Department, Hospital of Henares, Coslada, Madrid, Spain; 22 Internal Medicine Department, Infanta Cristina University Hospital, Parla, Madrid, Spain; 23 CIBER de Fisiopatología de La Obesidad Y Nutrición (CIBEROBN), Instituto de Salud Carlos III, Madrid, Spain

**Keywords:** COVID-19, SARS-CoV-2, Aged, 80 and over, Comorbidity, Morbidity, Mortality, Complications, Epidemiology, Spain

## Abstract

**Background:**

Old age is one of the most important risk factors for severe COVID-19. Few studies have analyzed changes in the clinical characteristics and prognosis of COVID-19 among older adults before the availability of vaccines. This work analyzes differences in clinical features and mortality in unvaccinated very old adults during the first and successive COVID-19 waves in Spain.

**Methods:**

This nationwide, multicenter, retrospective cohort study analyzes unvaccinated patients ≥ 80 years hospitalized for COVID-19 in 150 Spanish hospitals (SEMI-COVID-19 Registry). Patients were classified according to whether they were admitted in the first wave (March 1-June 30, 2020) or successive waves (July 1-December 31, 2020). The endpoint was all-cause in-hospital mortality, expressed as the case fatality rate (CFR).

**Results:**

Of the 21,461 patients hospitalized with COVID-19, 5,953 (27.7%) were ≥ 80 years (mean age [IQR]: 85.6 [82.3–89.2] years). Of them, 4,545 (76.3%) were admitted during the first wave and 1,408 (23.7%) during successive waves. Patients hospitalized in successive waves were older, had a greater Charlson Comorbidity Index and dependency, less cough and fever, and met fewer severity criteria at admission (qSOFA index, PO2/FiO2 ratio, inflammatory parameters). Significant differences were observed in treatments used in the first (greater use of antimalarials, lopinavir, and macrolides) and successive waves (greater use of corticosteroids, tocilizumab and remdesivir). In-hospital complications, especially acute respiratory distress syndrome and pneumonia, were less frequent in patients hospitalized in successive waves, except for heart failure. The CFR was significantly higher in the first wave (44.1% vs. 33.3%; -10.8%; *p* < 0.001) and was higher among patients ≥ 95 years (54.4% vs. 38.5%; -15.9%; *p* < 0.001). After adjustments to the model, the probability of death was 33% lower in successive waves (OR: 0.67; 95% CI: 0.57–0.79).

**Conclusions:**

Mortality declined significantly between the first and successive waves in very old unvaccinated patients hospitalized with COVID-19 in Spain. This decline could be explained by a greater availability of hospital resources and more effective treatments as the pandemic progressed, although other factors such as changes in SARS-CoV-2 virulence cannot be ruled out.

**Supplementary Information:**

The online version contains supplementary material available at 10.1186/s12877-022-03191-4.

## Background

More than 525 million infections and over 6.3 million deaths in the COVID-19 pandemic have been reported worldwide as of May 22, 2022 [1]. Starting in late February 2020, the SARS-CoV-2 epidemic spread rapidly throughout European countries, causing more than two million cases and 100,000 deaths on the continent in three months.

Social distancing measures and lockdowns were imposed in many European countries, including Spain, where a strict lockdown lasting 98 days was followed by a rapid reduction in COVID-19 cases [2]. After restrictions were lifted—and despite mass vaccination campaigns—countries around the world, including Spain, have experienced successive waves (local outbreaks) of infections. The response to these waves has been alternating intensification and relaxation of restrictions [3–10].

It has been firmly established that older adults were at highest risk of complications and death due to COVID-19 during the first wave [11–16]. There were considerable differences between old and very old patients in terms of inflammatory activity, disease severity, and adverse clinical outcomes [13, 16–20]. Indeed, the mortality rate was as high as 50% in hospitalized patients older than 80 years [13, 16, 17]. Multimorbidity, functional status, dementia, frailty, and long stays in residential care homes were potent prognostic markers of COVID-19 in older adults during the first wave [13, 16, 18–20].

The burden of disease on COVID-19 survivors, regardless of the severity of symptoms at disease onset, and on patients admitted to the intensive care unit as well as the rehabilitation needs associated with COVID-19 infection are receiving growing attention, increasing the level of evidence from studies which address these issues [21, 22]. Indeed, rehabilitation is very important in post-COVID-19 patients [23], as is the need for precise risk stratification in order to tailor optimal therapeutic interventions.

While the burden of the COVID-19 pandemic on patients has been widely recognized, the psychosocial impact of the working conditions healthcare professionals endured during the COVID-19 pandemic is also a crucial issue [23]. Recent works have pointed to the importance of a healthy work environment in ensuring job satisfaction among healthcare professionals and in order to avoid the burnout syndrome and role conflict experienced during the COVID-19 pandemic [24].

However, there is little information from large cohorts on changes in the patient profile and clinical outcomes in the different waves of the pandemic [25–27] and studies on very old patients hospitalized after the first wave are surprisingly scarce [28]. The aim of this study was to investigate differences in the clinical features and outcomes in patients ≥ 80 years hospitalized with COVID-19 during the first and successive waves in Spain prior to the availability of vaccines.

## Materials and methods

### Study design and population

This work is a retrospective cohort study in unvaccinated hospitalized patients ≥ 80 years of age with COVID-19 in Spain from March 1, 2020 to December 31, 2020. Data were drawn from the national SEMI-COVID-19 Registry. Vaccination against COVID-19 began in Spain in January 2021, but data on patient vaccination are not included in the registry. Therefore, as the aim was to study unvaccinated patients, no patients hospitalized after December 31, 2020 were analyzed in this study. Patients were grouped according to the pandemic wave in which they were included in the registry: the first wave (March 1 to June 30, 2020) or subsequent waves (July 1, 2020 to December 31, 2020). This cut-off date between the first and subsequent waves corresponds to the date used in several previous studies from Spain [5, 6, 8, 10].

### Definition of variables

All patient data were obtained from the SEMI-COVID-19 Registry of the Spanish Society of Internal Medicine, in which 150 Spanish hospitals participate. The SEMI-COVID-19 Registry prospectively collects data from the index admission of patients ≥ 18 years of age with COVID-19 microbiologically confirmed by reverse transcription polymerase chain reaction (RT-PCR) or antigen testing. More detailed information on the rationale, objectives, methodology, and preliminary results of the SEMI-COVID-19 Registry has recently been published [29].

The degree of dependence was assessed using the Barthel Index. Comorbidities were assessed using the age-adjusted Charlson Comorbidity Index (CCI) [30]. Patients were classified as having dyslipidemia, diabetes mellitus, or hypertension if they had a previous diagnosis on their electronic medical record (EMR) or were receiving pharmacological treatment for these conditions. Atherosclerotic cardiovascular disease was defined as a medical history of coronary artery disease (myocardial infarction, acute coronary syndrome, angina pectoris, or coronary revascularization), cerebrovascular disease (stroke, transient ischemic attack), or peripheral arterial disease (intermittent claudication, revascularization, lower limb amputation, or abdominal aortic aneurysm). Chronic lung disease was defined as a diagnosis of asthma and/or chronic obstructive pulmonary disease. Malignancy encompassed hematologic malignancy and/or solid tumors (excluding nonmelanoma skin cancer). Data on baseline comorbidities were collected from the EMR obtained from the hospitals.

Laboratory (blood gases, metabolic panel, complete blood count, coagulation) and imaging tests were performed on admission.

In-hospital complications included the presence of secondary bacterial pneumonia, acute respiratory distress syndrome (ARDS), acute heart failure, arrhythmia, acute coronary syndrome, myocarditis, seizures, stroke, shock, sepsis, acute renal failure, disseminated intravascular coagulation, venous thromboembolism, multiple organ dysfunction syndrome, and acute limb ischemia. Complications during hospitalization were defined pre-hoc and data on them were available in the EMR. Ventilatory support included invasive and noninvasive mechanical ventilation and high-flow oxygen therapy. Admissions within 30 days of hospital discharge were considered early readmissions.

Treatments used during hospitalization were classified as antimicrobial therapy (beta-lactams, macrolides, or quinolones), antiviral therapy (hydroxychloroquine, chloroquine, lopinavir/ritonavir, or remdesivir), immunomodulatory therapy (systemic corticosteroids, tocilizumab, baricitinib, or colchicine), or anticoagulant therapy (oral anticoagulants or low-molecular-weight heparin).

The primary endpoint of the study was all-cause in-hospital mortality, expressed as the case fatality rate (CFR), or the ratio of in-hospital deaths to the total number of patients hospitalized with COVID-19. Secondary endpoints were differences between waves in the clinical characteristics of patients on admission, medical treatments used during admission, and in-hospital complications.

### Statistical analysis

The characteristics of each group were analyzed using descriptive statistics. Continuous and categorical variables were expressed as medians and interquartile ranges (IQR) and as absolute values and percentages, respectively. Differences between groups were analyzed using the Mann–Whitney U test for continuous variables and Pearson's chi-square test for categorical variables. The 95% confidence interval (CI) for differences between the CFR in the first and successive waves was calculated using the methods of Newcombe et al. [31]. The significance of differences between the first and successive waves was calculated using odds ratio (OR) and the two-sample z-ratio. Time-to-event analyses were reported by means of Kaplan–Meier survival curves.

Three logistic regression models were used to analyze mortality: model A (adjusted for age, sex, degree of dependence, place of infection acquisition, qSOFA, and oxygen saturation), model B (adjusted for model A variables as well as use of corticosteroids, tocilizumab, and remdesivir), model C (adjusted for model A and model B variables as well as lymphocyte, lactate dehydrogenase, and C-reactive protein levels). Associations were expressed as adjusted OR and 95% CI. All analyses were performed using IBM SPSS Statistics for Windows, Version 25.0. Armonk, NY: IBM Corp. Statistical significance was defined as *p* < 0.05.

### Ethical aspects

This work was approved by the Institutional Research Ethics Committee of Málaga on March 27, 2020 (Ethics Committee code: SEMI-COVID-19 27–03-20), according to the guidelines of the Spanish Agency of Medicines and Health Products. All patients gave informed consent.

## Results

A total of 21,461 patients diagnosed with COVID-19 were included in the SEMI-COVID-19 Registry from March 1 to December 31, 2020: 17,123 in the first wave and 4,338 in successive waves. Of the total number of patients, 5,953 (27.7%) were ≥ 80 years of age and are the study population. In terms of waves, 26.5% (4,545/17,123) of patients admitted in the first wave and 32.5% (1,408/4,338) of patients admitted in successive waves were ≥ 80 years of age (Table 1).

**Table 1:** Total patients in the database and patients ≥ 80 years of age hospitalized with COVID-19 included in registry during the first and successive waves

	**First wave**	**Successive waves**	**Total**	***p* value**
Total patients in the SEMI-COVID-19 Registry database	17123	4338	21461	
Patients 10–79 years old	12578(73.5)	2930 (67.5)	15,508 (72.3)	<0.001
Patients ≥ 80 years old	4545 (26.5)	1408 (32.5)	5953 (27.7)	
80–84 years	1772 (10.3)	499 (11.5)	2271(10.6)	<0.001
85–89 years	1622 (9.5)	484 (11.2)	2106 (9.8)	<0.001
90–94 years	864 (5.0)	321 (7.4)	1185 (5.5)	<0.001
≥ 95 years	287 (1.7)	104 (2.4)	391 (1.8)	<0.001

### Epidemiological and clinical differences between waves (Table 2)

**Table 2:** Differences in demographic, and clinical findings on admission in patients ≥ 80 years hospitalized with COVID-19 during the first and successive waves

	**Total** **N (%)** **(** ***n*** ** = 5953)**	**First wave** **N (%)** **(** ***n*** ** = 4545)**	**Successive waves** **N (%)** **(** ***n*** ** = 1408)**	***p*** ** value**
Age, years, median (IQR),	85.6 (82.3–89.2)	85.5 (82.2–89.0)	86.1 (82.6–89.7)	** < 0.001**
Age, years, median (IQR),				**0.003**
80–84 years	2271 (38.1)	1772 (39.0)	499 (35.4)	
85–89 years	2106 (35.4)	1622 (35.7)	484 (34.4)	
90–94 years	1185 (19.9)	864 (19.0)	321 (22.8)	
≥ 95 years	391 (6.6)	287 (6.3)	104 (7.4)	
Sex, Male	3024 (50.8)	2331 (51.3)	693 (49.2)	0.175
**Acquisition**				
Community	4172 (70.2)	3170 (69.9)	1002 (70.2)	0.341
Nosocomial	426 (7.2)	330 (7.3)	96 (7.3)	0.492
Nursing Home	1036 (22.8)	1036 (22.8)	309 (22.0)	0.566
**Degree of dependence**				** < 0.001**
Independent or mild	3178 (56.2)	2516 (56.4)	662 (47.2)	
Moderate	1486 (54.3)	1076 (24.1)	410 (29.2)	
Severe	1201 (20.5)	871 (19.5)	330 (23.5)	
**Comorbidities**				
Baseline CCI, median (IQR)	6 (5–7)	6 (5–7)	6 (5–7)	**0.007**
Baseline CCI ≥ 6, n (%)	2445 (42.1)	1892 (42.9)	553 (39.8)	**0.006**
Hypertension	4516 (76.0)	3412 (75.2)	1104 (78.4)	**0.014**
Non-atherosclerotic cardiovascular diseases^a^	1918 (32.3)	1464 (32.3)	454 (32.3)	0.993
Atherosclerotic cardiovascular diseases^b^	1750 (29.6)	1320 (29.2)	431 (30.7)	0.306
Dementia	1671 (28.1)	1269 (28.0)	402 (28.8)	0.678
Diabetes mellitus	1645 (27.7)	1204 (26.6)	441 (31.3)	** < 0.001**
Chronic pulmonary disease^c^	1144 (19.3)	850 (18.8)	294(20.9)	0.079
Obesity^f^	893 (16.8)	675 (16.7)	218 (17.3)	0.584
Malignancy^d^	806 (13.6)	615 (13.6)	191 (13.6)	0.993
Moderate-to-severe kidney disease^e^	692 (11.7)	521 (11.5)	171 (12.1)	0.476
**Symptoms**				
Duration of symptoms in days, median (IQR)	5 (2–7)	5 (2–7)	4 (2–7)	**0.029**
Fever	4158 (70.2)	3338 (73.9)	820 (58.3)	** < 0.001**
Dyspnea	3621 (61.1)	2757 (61.0)	864 (61.4)	0.791
Cough	3537 (59.6)	2791 (61.7)	746 (53.1)	** < 0.001**
Asthenia	2326 (39.6)	1696 (38.0)	630 (44.7)	** < 0.001**
Confusion	1644 (27.8)	1265 (28.1)	379 (27.0)	0.428
Anorexia	1317 (22.5)	963 (21.6)	354 (25.1)	**0.006**
Diarrhea	975 (16.5)	732 (16.3)	243 (17.3)	0.396
Arthralgia-myalgias	983 (16.7)	774 (17.3)	209 (14.9)	**0.032**
Vomiting	379 (6.4)	287 (6.4)	92 (6.5)	0.855
Abdominal pain	320 (5.4)	255 (5.7)	65 (4.6)	0.118
Odynophagia	330 (5.6)	272 (6.1)	58 (4.1)	**0.005**
Headache	310 (5.3)	238 (5.3)	72 (5.1)	0.747
Ageusia	183 (3.1)	145 (3.3)	38 (2.7)	0.275
Anosmia	158 (2.7)	127 (2.9)	31 (2.2)	0.173
**Physical examination**				
Oxygen saturation ≤ 94%	3236 (51.4)	2334 (52.7)	902 (48.3)	**0.001**
Temperature ≥ 37.8 ºC	1025 (16.7)	830 (19.4)	195 (10.6)	** < 0.001**
Hypotension	467 (7.5)	334 (7.6)	138 (7.1)	0.471
Tachycardia	1203 (19.2)	881 (20.0)	322 (17.2)	**0.009**
Tachypnea	2314 (39.6)	1810 (40.8)	504 (36.0)	**0.001**
Pulmonary rhonchi	1087 (17.6)	847 (19.1)	240 (17.7)	0.218
qSOFA index ≥ 2	1076 (16.7)	798 (17.6)	278 (14.6)	**0.004**

*CCI* Charlson Comorbidity Index, *IQR* Interquartile range, *N* (%) Number of cases (percentage), *qSOFA* Quick sequential organ failure assessment

^a^Non-atherosclerotic heart disease includes atrial fibrillation and/or heart failure. ^b^Atherosclerotic cardiovascular disease includes coronary, cerebrovascular, and/or peripheral vascular disease. ^c^Chronic pulmonary disease includes chronic obstructive pulmonary diseases and/or asthma. ^d^Malignancy includes solid tumors or hematological neoplasia. ^e^Kidney disease is defined as an estimated glomerular filtration rate (eGFR) < 45 mL/min/1.73 m^2^ according to the CKD-EPI equation. ^f^Obesity is defined as a body mass index > 30 kg/ m^2^

Statistically significant differences are indicated in bold

The median age (IQR) of patients ≥ 80 years of age was 85.6 (82.3–89.2) years and 50.8% were male. Patients hospitalized during successive waves had higher rates of moderate and severe dependence compared to patients hospitalized during the first wave (moderate dependence in successive waves: 29.2% vs. first wave: 24.1%; severe dependence in successive waves: 23.5% vs. first wave: 19.5%; *p* < 0.001).

The median CCI was 6 and was slightly higher in successive waves (CCI ≥ 6: 19.5% vs. 23.5%, *p* < 0.001). The rates of comorbidities were similar in the two periods, except for hypertension (75.2% vs. 78.4%, *p* = 0.014) and diabetes mellitus (26.6% vs. 31.1%, *p* < 0.001), which were lower in the first wave.

The duration of symptoms before admission was shorter in successive waves compared to the first wave (median [IQR]: 4 [2-7] vs. 5 [2-7] days, *p* = 0.021). Patients admitted in the second wave had more fever (73.9% vs. 58.3%, *p* < 0.001), cough (61.7% vs. 53.1%, *p* < 0.001), arthralgias-myalgias (17.3% vs. 14.9%; *p* = 0.032), and odynophagia (6.1% vs. 3.8%; *p* = 0.005) and fewer presented with asthenia (38.0% vs. 44.7%, *p* < 0.001) and anorexia (21.6% vs. 25.1%, *p* = 0.006) than patients in the first wave.

Upon physical examination, patients admitted in successive waves had less hypoxemia (52.7% vs. 48.3%; *p* < 0.001), fever (19.4% vs. 10.6%, *p* < 0.001), tachypnea (40.8% vs. 36%, *p* = 0.001), and qSOFA ≥ 2 (17.6% vs. 14.6%, *p* = 0.004).

### Radiological, and analytical differences between waves (Table 3)

**Table 3:** Radiological, and analytical findings on admission in patients ≥ 80 years hospitalized with COVID-19 during the first and successive waves

	**Total** **N (%)** **(** ***n*** ** = 5953)**	**First wave** **N (%)** **(** ***n*** ** = 4545)**	**Successive waves** **N (%)** **(** ***n*** ** = 1408)**	***p*** ** value**
**Chest X-ray findings**				** < 0.001**
Normal	1091 (18.7)	768 (17.3)	323 (23.1)	
Unilateral infiltrates	1168 (20.0)	867 (19.5)	301 (21.5)	
Bilateral infiltrates	3584 (61.3)	2807 (63.2)	777 (55.5)	
**Laboratory findings**				
**Arterial blood gases**				
PO_2_/FiO_2_ ratio	277 (222–327)	273 (214–323)	289 (242–333)	** < 0.001**
**Blood count & biochemistry**				
Lymphocytes (× 10^3^/μL)	0.88 (0.06–1.23)	0.88 (0.60–1.23)	0.87 (0.6–1.23)	0.532
Lactate dehydrogenase (U/L)	322 (246–448)	330 (251–463)	302 (233–412)	** < 0.001**
C-reactive protein (mg/L)	71.0 (24.0–138)	71.6 (23.5–143)	69.8 (27.8–128)	0.330
D-dimer (ng/mL)	1.01 (0.55–2.02)	1.02 (0.54–2.02)	1.00 (0.55–2.01)	0.558
Serum ferritin (μg/L)	445 (212–953)	484 (230–1013)	396 (189–856)	** < 0.001**
Fibrinogen (mg/L)	598 (500–700)	607 (500–706)	564 (461–700)	** < 0.001**

Statistically significant differences are indicated in bold

There were fewer cases of bilateral infiltrates on a chest x-ray upon admission in patients in successive waves (63.2% vs 55.5%, *p* < 0.001). In regard to analytical parameters, patients admitted in successive waves had lower levels of inflammatory parameters than patients in the first wave, including lactate dehydrogenase (330 [251–463] vs. 302 [233–412], *p* < 0.001), serum ferritin (484 [230–1013] vs. 396 [189–856], *p* < 0.001), and fibrinogen (607 [500–706] vs. 564 [461–700], *p* < 0.001). There were no differences in lymphocyte, C-reactive protein, and D-dimer values between the first and successive waves, but the PO2/FiO2 ratio was higher in successive waves (273 [214–323] vs. 289 [242–333], *p* < 0.001).

### Treatment and complications between waves (Table 4)

**Table 4:** Differences in treatment, complications, and outcomes in Patients ≥ 80 Years Hospitalized with COVID-19 During the First and Successive Waves

	**Total** **N (%)** **(** ***n*** ** = 5953)**	**First wave** **N (%)** **(** ***n*** ** = 4545)**	**Successive waves** **N (%)** **(** ***n*** ** = 1408)**	***p*** ** value**
**Immunomodulatory therapy**				
Systemic corticosteroids	2902 (49.0)	1770 (39.2)	1135 (80.6)	** < 0.001**
Tocilizumab	228(3.8)	151 (3.3)	77 (5.5)	** < 0.001**
Colchicine	68 (1.2)	64 (1.4)	4 (0.3)	** < 0.001**
Anakinra	31 (0.5)	21 (0.5)	10 (0.7)	0.270
Baricitinib	26 (0.5)	21 (0.6)	5 (048)	0.360
**Antivirals**				
Hydroxychloroquine	3467 (58.5)	3463 (76.5)	4 (0.3)	** < 0.001**
Lopinavir/ritonavir	1893 (31.9)	1885 (41.7)	8 (0.6)	** < 0.001**
Interferon	302 (5.1)	302 (6.7)	0 (0.0)	** < 0.001**
Remdesivir	165 (2.8)	7 (0.2)	158 (11.2)	** < 0.001**
Chloroquine	160 (2.7)	160 (3.5)	0 (0.0)	** < 0.001**
Immunoglobulin	10 (0.2)	10 (0.2)	0 (0.0)	0.131
**Antibiotics**				
Beta-lactams	4300 (72.6)	3307 (73.2)	993 (70.7)	**0.064**
Quinolones	942 (16.0)	689 (15.3)	253 (18.0)	**0.017**
Macrolides	3060 (51.7)	2522 (55.9)	538 (33.3)	** < 0.001**
**Ventilatory therapy**				
High-flow nasal cannula oxygen	398(6.7)	312 (6.9)	86 (6.1)	0.288
Non-invasive mechanical ventilation	249 (4.2)	185 (4.1)	64 (4.6)	0.460
Invasive mechanic ventilation	70 (1.2)	55 (1.2)	15 (1.1)	0.642
**Anticoagulant therapy**				
Oral anticoagulants ^a^	428 (7.2)	323 (7.2)	105 (7.5)	0.681
Low-molecular-weight heparin	425 (83.5)	3678 (81.5)	1247 (88.8)	** < 0.001**
**Complications**				
ARDS, severe	1932 (32.6)	1613 (35.7)	319 (22.7)	** < 0.001**
Acute kidney failure	1370 (23.1)	1059 (23.4)	311 (22.1)	0.324
Acute heart failure	794 (13.4)	560 (12.4)	234 (16.6)	** < 0.001**
Pneumonia	795 (13.4)	649 (14.3)	146 (10.4)	** < 0.001**
Multiple organ dysfunction syndrome	529 (8.9)	440 (9.7)	89 (6.3)	** < 0.001**
Sepsis	450 (7.6)	366 (8.1)	84 (6.0)	**0.009**
Arrhythmia	382 (6.4)	283 (6.3)	99 (7.0)	0.297
Shock	229 (3.9)	186 (4.1)	43 (3.1)	0.073
Venous thromboembolism	110 (1.9)	79 (1.7)	43 (3.1)	0.172
Acute coronary syndrome	84 (1.4)	63 (1.4)	21 (1.2)	0.270
Stroke	54 (0.9)	43 (0.9)	11 (0.8)	0.562
Myocarditis	52 (0.9)	37 (0.8)	15 (1.1)	0.381
Intravascular coagulation	61 (1.0)	52 (1.1)	9 (0.6)	0.098
Epileptic seizures	44 (0.7)	35 (0.8)	9 (0.6)	0.614
Acute peripheral ischemic	36 (0.6)	30 (0.7)	6 (0.4)	0.315
**Outcomes**				
Intensive care unit admission	111 (1.9)	81 (1.8)	30 (1.1)	0.400
Readmission	341 (5.9)	227 (5.2)	114 (8.1)	** < 0.001**
Death	2697 (41.8)	2004 (44.1)	693 (36.4)	** < 0.001**
**Days of hospitalization, median (IQR)**	14 (10–21)	14 (9–21)	14 (10–21)	0.734

*N* (%) Number of cases (percentage), *ARDS* Acute respiratory distress syndrome, *IQR* Interquartile range:

^a^Anticoagulant therapy (dicumarin or direct anticoagulant)

Statistically significant differences are indicated in bold

The use of hydroxychloroquine/chloroquine (8.0% vs. 0.3%, *p* < 0.001), lopinavir/ritonavir (41.7% vs. 0.6%, *p* < 0.001), interferon (6.7% vs. 0.0%, *p* < 0.001), and macrolides (55.9% vs. 33.3%, *p* < 0.001) was significantly higher in the first wave. In contrast, during successive waves, the use of corticosteroids (39.2% vs. 80.6%, *p* < 0.001), remdesivir (0.2% vs. 11.2%, *p* < 0.001), tocilizumab (3.3% vs. 5.5%, *p* < 0.001), and low-molecular-weight heparin (81.5% vs. 88.8%, *p* < 0.001) increased significantly. No differences were found in the indication for ventilatory support between the two periods analyzed.

The use of high-flow nasal cannula oxygen, non-invasive mechanical ventilation, and invasive mechanic ventilation was not common and there were no differences between the first and successive waves (6.9% vs. 6.1%, 4.1% vs. 4.6% and 1.2% vs. 1.1%, respectively).

In general, patients admitted after the first wave had fewer complications, especially severe ARDS (35.7% vs. 22.7%, *p* < 0.001), bacterial pneumonia (14.3% vs. 10.4%, *p* < 0.001), sepsis (8.1% vs. 5.6%, *p* < 0.001), and multiple organ dysfunction syndrome (8.1% vs. 6%, *p* < 0.001). However, cases of acute heart failure were more frequent in the successive waves (12.4% vs. 16.6%, *p* < 0.001).

### Outcomes between waves (Table 5)

**Table 5:** Case-fatality rate (CFR) in patients ≥ 80 years of age hospitalized with COVID-19

	**No. of deaths/Total No. of Patients**	**% of total deaths**	**CFR %**	**OR (95% CI)**	***p*** ** value**
**Total**					
80–84 years	818/2271	33.1	36.0	Ref	
85–90 years	921/2106	37.2	43.7	1.38 (1.22–1.55)	** < 0.001**
90–94 years	538/1185	21.8	45.4	1.47 (1.38–1.70)	** < 0.001**
≥ 95 years	196/391	7.9	50.1	1.78 (1.43–2.21)	** < 0.001**
Total	2473/5953	100	41.5		
**First wave**					
80–84 years	685/1772	34.2	38.7	Ref	
85–90 years	750/1622	37.4	46.2	1.36 (1.19–1.56)	** < 0.001**
90–94 years	413/864	20.6	47.8	1.45 (1.23–1.71)	** < 0.001**
≥ 95 years	156/287	7.8	54.4	1.89 (1.47–2.42)	** < 0.001**
Total	2004/4545	100	44.1		
**Successive waves**					
80–84 years	133/499	28.4	26.7	Ref	
85–90 years	171/484	36.5	35.3	1.50 (1.14–1.97)	**0.003**
90–94 years	125/321	26.7	38.9	1.75 (1.30–2.37)	** < 0.001**
≥ 95 years	40/104	8.5	38.5	1.72 1.10–2.67)	**0.016**
Total	469/1408	100	33.3	-	
Differences between first and successive waves*				95% (CI)*	***p*** value**
80–84 years	-	-	-	-12.0 (-7.4; -16.4)	** < 0.001**
85–90 years	-	-	-	-10.9 (-5.9; -13.2)	** < 0.001**
90–94 years	-	-	-	-8.9 (-2.5, -15.0)	**0.006**
≥ 95 years	-	-	-	-15.8 (-4.7; -26.3)	** < 0.001**
Total	-	-	-	-10.8 (-7.9; -13.6)	** < 0.001**

^*^The confidence intervals for differences between CFR in the first wave and successive waves were calculated using the methods of Newcombe et al. ** Significance of differences between the first and successive wave was calculated using the two proportion z-test

*OR* Odds ratio, *CI* confidence interval

Statistically significant differences are indicated in bold

The CFR was significantly higher in the first wave than in successive waves (44.1% vs 33.3%, *p* < 0.001) (CFR difference: -10.8 [95% CI: -7.5 to -13.6], *p* < 0.001). The CFR was significantly lower in all age ranges in patients hospitalized after the first wave, but this reduction in mortality was especially notable in patients ≥ 95 years (-15.8, 95% CI: -4.7 to -26.3, *p* < 0.001). The probability of death in successive waves was 37% lower than in the first wave (OR: 0.63; 95% CI: 0.55–0.72) without adjusted.

The risk of mortality in those hospitalized after the first wave remained lower even after adjusting for age, sex, degree of dependence, place of infection acquisition, qSOFA, and oxygen saturation (Model A) (OR: 0.61, 95% CI: 0.53–0.70, *p* < 0.001). It was also lower after adjusting for model A variables as well as corticosteroid, tocilizumab, and remdesivir use (Model B) (OR: 0.55, 95% CI: 0.47–0.64, *p* < 0.001) and after adjusting for model A variables, model B variables, and lymphocyte, lactate dehydrogenase, and C-reactive protein levels (Model C) (OR: 0.6, 95% CI: 0.57–0.79, *p* < 0.001) (Table 6).

**Table 6:** Multivariable logistic regression model for in-hospital mortality in patients ≥ 80 years of age hospitalized with COVID-19

	**Model A**^**a**^		**Model B**^**b**^		**Model C**^**c**^	
	**OR (95% CI)**	***p* value**	**OR (95% CI)**	***p* value**	**OR (95% CI)**	***p* value**
**Wave**						
First	Ref		Ref	-	Ref	
Successive	0.61 (0.53–0.70)	< 0.001	0.58 (0.50–0.68)	< 0.001	0.67 (0.57–0.79)	< 0.001
**Age group**						
80–84 years	Ref		Ref		Ref	
85–90 years	1.25 (1.09–1.43)	0.001	1.26 (1.10–1.44)	0.001	1.31 (1.25–1.54)	0.001
90–94 years	1.27 (1.08–1.50)	0.004	1.30 (1.10–1.53)	0.001	1.38 (1.13–1.67)	0.001
≥ 95 years	1.41 (1.11–1.87)	0.003	1.47 (1.14–1.90)	0.003	1.56 (1.17–2.09)	0.003
Sex, male	1.44 (1.28–1.63)	< 0.001	1.41 (1.25–1.59)	< 0.001	1.33 (1.16–1.53)	< 0.001
**Acquisition**						
Community	Ref	-	Ref	-	Ref	
Nosocomial	1.52 (1.21–1.90)	< 0.001	1.54 (1.23–1.94)	< 0.001	1.46 (1.11–1.92)	< 0.001
Nursing Home	0.71 (0.61–0.84)	< 0.001	0.71 (0.61–0.84)	< 0.001	0.72 (0.61–0.88)	< 0.001
**Degree of dependence**						
Independent or mild	Ref	-	Ref	-	Ref	
Moderate	1.40 (1.21–1.64)	< 0.001	1.42 (1.21–1.63)	< 0.001	1.5 (1–29-1.81)	< 0.001
Severe	1.63 (1.37–1.94)	< 0.001	1.67 (1.40–2.00)	< 0.001	2.05 (1.67–2.53)	< 0.001
**Comorbidities**						
CCI	1.07 (1.04–1.10)	< 0.001	1.07 (1.04–1.10)	< 0.001	1.07 (1.03–1.11)	< 0.001
**Physical examination**						
Oxygen saturation < 94%	2.15 (1.91–2.41)	< 0.001	2.09 (1.87–2.35)	< 0.001	1.58 (1.38–1.81)	< 0.001
qSOFA score ≥ 2	2.79 (2.38–3.27)	< 0.001	2.09 (1.86–2.25)	< 0.001	2.31 1.92–2.78)	< 0.001
**Treatment**						
Steroid	-	-	1.29 (1.13–1.45)	< 0.001	1.29 (1.12–1.50)	< 0.001
Tocilizumab			1.35 (1.00–1.84)	0.049	1.23 (0.89–1.71)	0.68
Remdesivir			0.52 (0.34–0.79)	0.002	0.51 (0.32–7.98)	0.509
**Laboratory findings**						
Lymphocytes (× 10^3^/μL)					1.00 (1.00–1.00)	< 0.001
Lactate dehydrogenase (U/L)					1.00 (1.00–1.00)	< 0.001
C-reactive protein (mg/L)					1.00 (1.00–1.00)	< 0.001

*CCI* Charlson Comorbidity Index, *OR* Odds ratio, *CI* Interval confidence, *qSOFA* quick sequential organ failure assessment; *Ref* Reference

^a^Model A. Adjusted for age group, sex, place of acquisition, degree of dependence, baseline Charlson Comorbidity Index, oxygen saturation, and qSOFA score

^b^Model B. Adjusted for age group; sex; place of acquisition; degree of dependence; baseline Charlson Comorbidity Index; oxygen saturation; qSOFA score; and treatment with steroids, tocilizumab, and remdesivir

^c^Model C. Adjusted for age group; sex; place of acquisition; degree of dependence; baseline Charlson Comorbidity Index; oxygen saturation; qSOFA score; treatment with steroids, tocilizumab, and remdesivir; and laboratory findings of lymphocytes, lactate dehydrogenase, and C-reactive protein

**Fig. 1:**
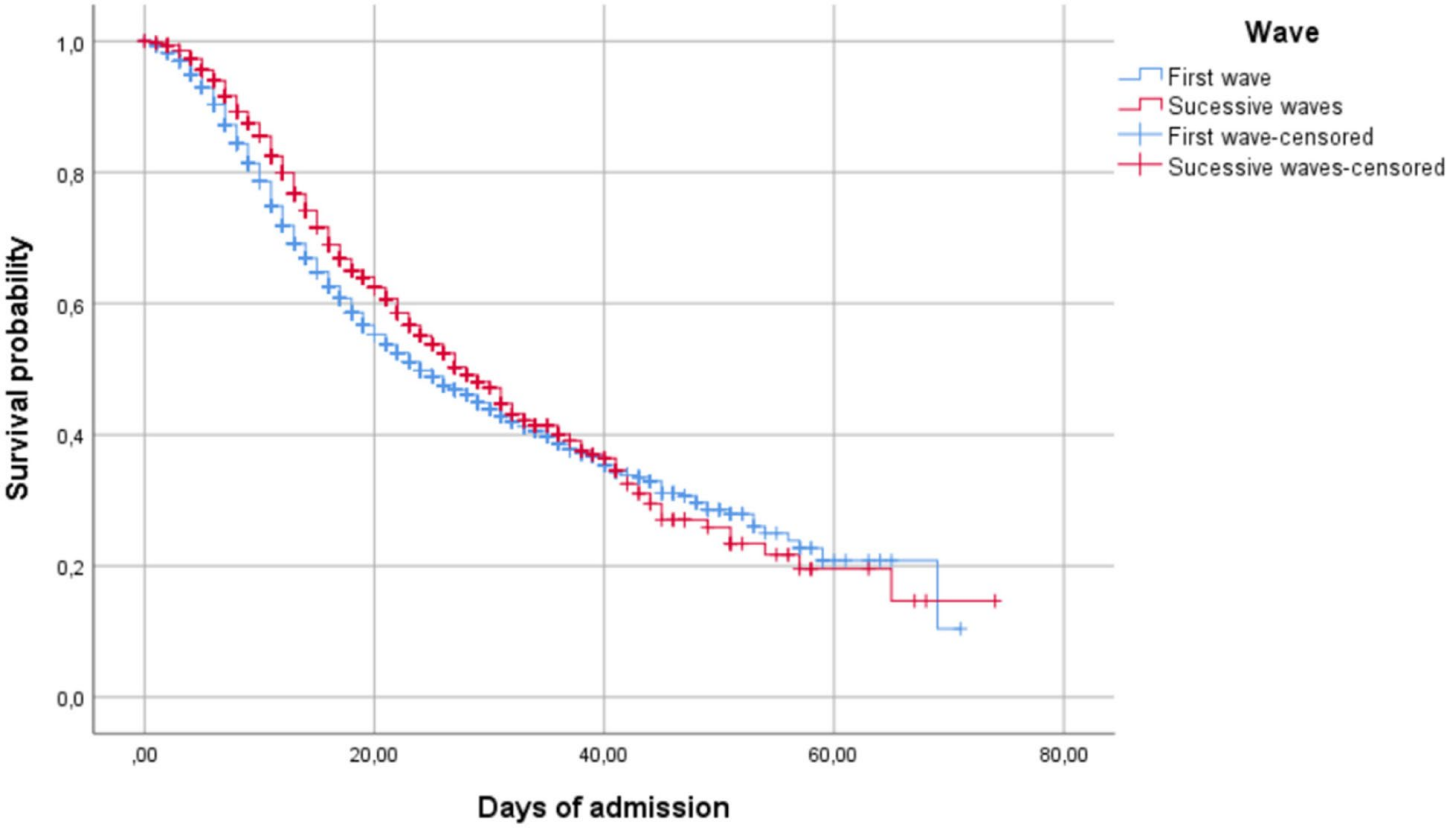
Probability of survival in the first and successive waves

**Fig. 2:**
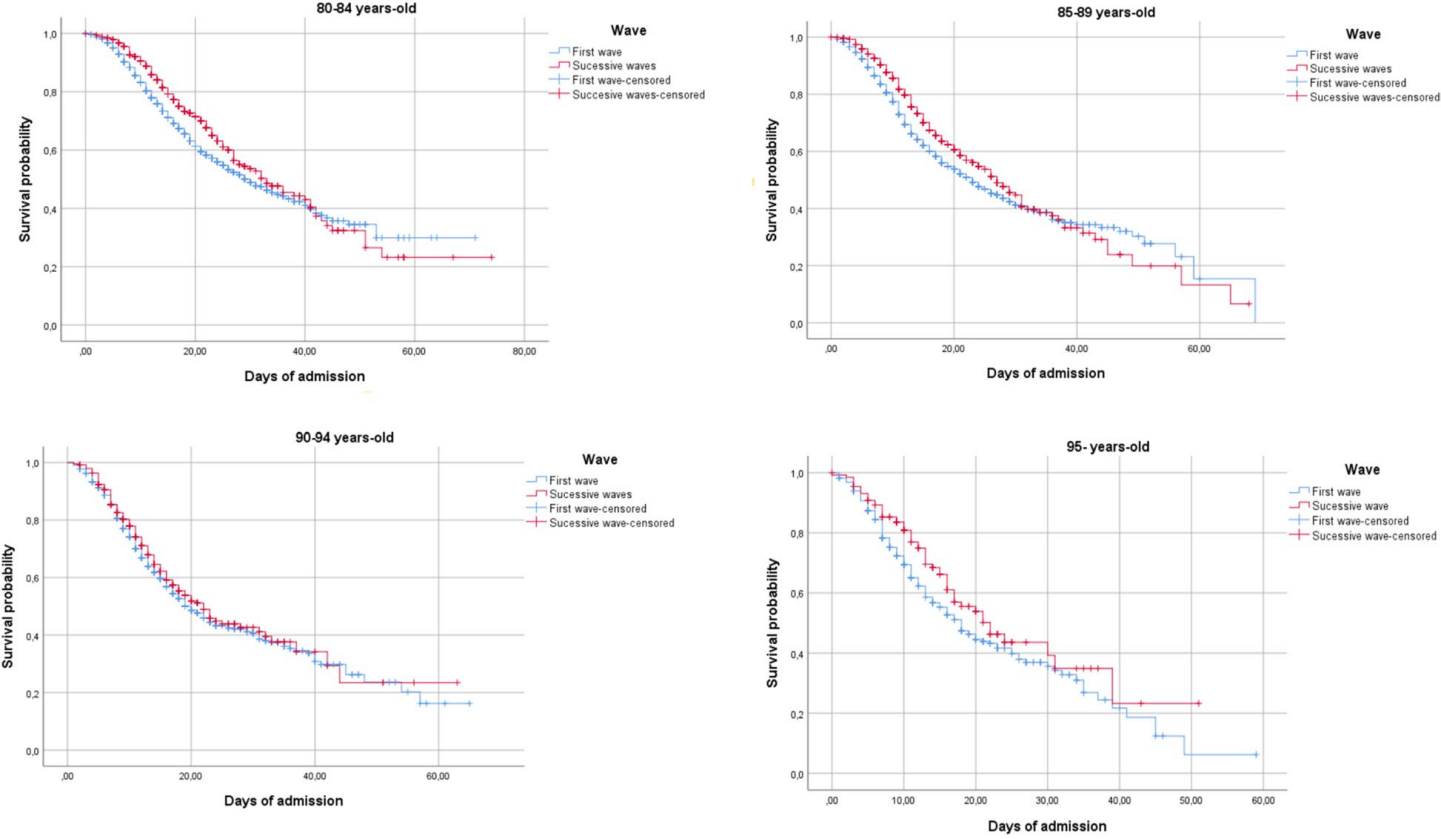
Probability of survival in the first and successive waves by age group (80–84, 85–89, 90–94, and 95 years)

Figure 1 shows the probability of survival in the first and successive waves (log rank, *p* < 0.001) and Fig. 2 shows the probability of survival for older adults in the first and successive waves (log rank, *p* < 0.001) according to age group (80–84, 85–89, 90–94, and 95 years).

There were no differences between waves in length of hospitalization (median [IQR] 14 [10-21] vs. 14 [10-21] days, *p* = 0.73) or ICU admission (1.8% vs. 1.1%, *p* = 0.4) but readmission was more frequent in successive waves (5.2% vs. 8.1%, *p* < 0.001).

## Discussion

In this large cohort of very old patients hospitalized with COVID-19 in Spain, we found a significant reduction of nearly 40% in in-hospital mortality after the first wave of the pandemic. This reduction in mortality cannot be explained solely by differences in the main predictors of mortality previously described in this population (age, functional status, dementia, comorbidities) [11–16] among those admitted in the first and successive waves because our models controlled for these possible confounding variables. Therefore, our findings suggest that this reduction in mortality must be explained by other factors, such as a greater availability of hospital resources after the first wave, during which hospital systems were overloaded and resources were in short supply, and improved medical management during hospitalization.

Previous works have reported a reduction in all-cause mortality in patients hospitalized with COVID-19 after the first wave in other settings, including in the United Kingdom, Italy, and the United States of America [7, 32–34]. However, other studies in Spain and in other European and Latin American countries have not reported a reduction in mortality after the first wave [8–10, 35]. To the best of our knowledge, this is the first study that analyzes differences in mortality in very old patients between pandemic waves in Spain**.**

Theoretically, the mortality rate of COVID-19 depends on patient-related factors, medical management factors, and virus-dependent factors. In regard to patient-related factors, advanced age has been identified as one of the strongest predictors of mortality since the beginning of the pandemic [29, 36–42]. In addition to old age, risk factors for poor prognosis have been reported in older adult patients hospitalized for COVID-19, including functional status and dementia [11–16]. In our study, we found no differences in age, the degree of comorbidity, or the rate of dementia between older adult patients hospitalized in the first and successive waves. Moreover, the older adult patients admitted in successive waves in our series had a worse functional status than patients hospitalized during the first wave and it is well-known that dependence is a strong predictor of poor prognosis in older adults with COVID-19 [16]. Therefore, it does not seem plausible that patient-related factors would be able to explain the lower mortality rate observed among those hospitalized in successive waves in our study.

In regard to medical management factors, our study reflects how the use of medical therapies for severe COVID-19 has changed throughout the pandemic. In the first wave, more than 80% of hospitalized patients were treated with hydroxychloroquine or chloroquine, nearly 60% with macrolides, and more than 40% with lopinavir. The prescribing of all these therapies declined sharply in successive waves when the results of clinical research trials that did not support their efficacy were published [43–46]. Instead, during successive waves, more evidence-based treatments such as corticosteroids [46], tocilizumab [47], and remdesivir [48] were indicated. In addition, though the use of anticoagulant drugs, mainly low-molecular-weight heparin, was already high in the first wave (86.7%), its use became almost universal in successive waves (97.4%), reflecting adherence to recommendations advising their use in severe COVID-19 [46]. On the other hand, the use of beta-lactams declined significantly after the first wave. Excessive use of inappropriate empirical antibiotic therapy during the first wave of COVID-19 has been cautioned against, considering that the rate of bacterial coinfection in these patients is low [49–51]. Overall, this trend toward using therapies with proven benefit may have contributed to reducing both complications and in-hospital mortality after the first wave of COVID-19. It is noteworthy that in our study, the greatest reduction in mortality during the pandemic was found in patients aged ≥ 95 years. This shows that even very old patients can benefit from intensive in-hospital management if their overall condition allows for it [16].

The risk factors associated with increased mortality in our study are the same as those previously reported in very old patients [29, 36–42]. Aging, moderate-severe dependence, CCI, and clinical severity on admission (oxygen saturation < 94%, qSOFA score 2) have been associated with poor outcomes in older patients hospitalized with COVID-19 [29, 36–42]. The increased mortality associated with corticosteroid use may be explained by the fact that it is more frequently use in severe cases [52–55]. Finally, compared to community-acquired infection, patients with nosocomial COVID-19 had a higher mortality rate, a finding that has been previously reported [56]. On the other hand, we observed a lower in-hospital mortality rate in older patients with COVID-19 who acquired the infection in long-term care facilities compared with patients with community-acquired disease. This finding has previously been described and explained by the possible earlier identification and treatment of COVID-19 symptoms as well as earlier hospitalization of these patients [57].

### Limitations

Our study has some limitations. First, due to its observational design, it is not possible to establish causality. Second, we cannot exclude undetected bias in our analysis, either because of limitations in the assessment of functional status or because of changes in admission criteria during the pandemic. On the one hand, we only calculated the Barthel Index, since the clinical condition of the patients prevented a more exhaustive geriatric assessment; thus, our registry lacks data on some geriatric syndromes (falls, delirium, malnutrition, etc.). Third, the it is plausible that, in the context of an overloaded hospital system with limited availability of resources, only older adult patients with a good functional status were admitted during the first wave whereas more relaxed admission criteria were followed during the following waves, when the pressure on hospitals was lower. In support of the latter argument, we found that patients admitted after the first wave showed fewer clinical and laboratory criteria of severity and had a shorter duration of symptoms than patients hospitalized in the first wave. Fourth, we do not have data on the SARS-CoV-2 strains of patients hospitalized with COVID-19 and cannot rule out that the reduction in complications and mortality observed after the first wave may be at least partially explained by a lower virulence of SARS-CoV-2 in successive waves [57]. Finally, this study is limited to unvaccinated patients. Therefore, our conclusions are not able to be extrapolated to vaccinated populations.

## Conclusions

In conclusion, we found a significant reduction in complications and mortality in older patients hospitalized with COVID-19 after the first wave in Spain. Our data suggest that both a greater availability of hospital resources and the use of more effective medical therapies may explain this improvement, although a possible reduction in SARS-CoV-2 virulence during successive waves cannot be ruled out.

Mortality in elderly patients declined after the first wave. This may have been due to a better understanding of the disease and the use of targeted treatments such as steroids in patients with hypoxemia. Going forward, precise risk stratification is needed in order to tailor optimal therapeutic interventions and continue to reduce SARS-CoV-2 mortality in elderly patients.

## Supplementary Information

**Additional file 1.** List of the SEMI-COVID-19 Network members.

## Data Availability

The datasets generated during the current study are not publicly available due data are not publicly available due to privacy or ethical restrictions, but are available from the corresponding author on reasonable request.

## Abbreviations

ARDS: Acute respiratory distress syndrome
CCI: Charlson Comorbidity Index
CFR: Case fatality rate
COVID-19: Coronavirus disease 2019
EMR: Electronic medical record
IQR: Interquartile ranges
OR: Odds ratio
qSOFA: Quick sequential organ failure assessment
SARS-CoV-2: Severe acute respiratory syndrome coronavirus 2
